# Arbitrarily tunable orbital angular momentum of photons

**DOI:** 10.1038/srep29212

**Published:** 2016-07-05

**Authors:** Yue Pan, Xu-Zhen Gao, Zhi-Cheng Ren, Xi-Lin Wang, Chenghou Tu, Yongnan Li, Hui-Tian Wang

**Affiliations:** 1 MOE Key Laboratory of Weak Light Nonlinear Photonics and School of Physics, Nankai University, Tianjin 300071, China; 2 National Laboratory of Solid State Microstructures, Nanjing University, Nanjing 210093, China; 3 Collaborative Innovation Center of Advanced Microstructures, Nanjing University, Nanjing 210093, China

## Abstract

Orbital angular momentum (OAM) of photons, as a new fundamental degree of freedom, has excited a great diversity of interest, because of a variety of emerging applications. Arbitrarily tunable OAM has gained much attention, but its creation remains still a tremendous challenge. We demonstrate the realization of well-controlled arbitrarily tunable OAM in both theory and experiment. We present the concept of general OAM, which extends the OAM carried by the scalar vortex field to the OAM carried by the azimuthally varying polarized vector field. The arbitrarily tunable OAM we presented has the same characteristics as the well-defined integer OAM: intrinsic OAM, uniform local OAM and intensity ring, and propagation stability. The arbitrarily tunable OAM has unique natures: it is allowed to be flexibly tailored and the radius of the focusing ring can have various choices for a desired OAM, which are of great significance to the benefit of surprising applications of the arbitrary OAM.

Besides spin angular momentum (SAM) associated with circular polarization, with two possible quantized values of ±*ħ*, a scalar (*homogeneously polarized*) vortex light field with a helical phase of exp(*jmϕ*) could also carry an intrinsic and eigen orbital angular momentum (OAM) of *mħ* per photon^1^. The photon OAM, as a new fundamental controlling degree of freedom and infinite quantum states of photons, has intrigued a broad interest^2^,^3^, due to its important applications in a variety of realms^4^,^5^,^6^,^7^,^8^,^9^,^10^,^11^,^12^,^13^,^14^. The concept of the photon OAM has been extended to other waves such as electron beams^15^, x ray^16^, radio wave^17^, and matter wave^18^. The photon OAM is undoubtedly an extensively interesting topic and brings surprisingly the recent exploitations.

The fractional OAM has been given evermore increasing attention^19^,^20^,^21^,^22^,^23^,^24^,^25^, due to its theoretical significance and its novel applications. Differently from a scalar vortex light field carrying the integer OAM, the light field carrying the fractional OAM will in general undergo an evolution during its propagation in free space, resulting in that its local OAM and its intensity pattern exhibit both the azimuthal nonuniformity and even its propagation is unstable. Such a kind of fractional OAM is a combination of a series of weighted integer OAMs. Regardless of its evolving intensity pattern and vortex structure, the OAM integrated over the whole light field cross section is still invariant during its propagation, which reflects the topologically invariant nature of the fractional OAM. It is always expected that the arbitrarily tunable OAM, like the integer OAM, not only is continuously tunable, but also has the uniformity in both the local OAM and the intensity ring, due to its important applications such as quantum entanglement and optical micro-manipulation (for instance, the motion speed of the trapped microparticles is required to be continuously changeable). However, creating and tailoring the arbitrarily tunable OAM meets still a tremendous challenge.

The attempts at creating the photon OAM almost focuses *only* on the *scalar* light fields so far. The integer OAM states can be used to define an infinitely dimensional discrete Hilbert space. Since the photons have two orthogonally polarized modes, one can also define another orthogonally polarized infinitely dimensional discrete Hilbert space. To get the substantial progress on the photon OAM, the most possible solution may be to break through the limit of scalar light fields, by employing the *vector* light fields^26^,^27^,^28^. Here we report the realization of well-controlled arbitrarily tunable OAM, based on the vector fields. We present the concept of general OAM, which extends the OAM carried by the scalar vortex field to the OAM carried by the azimuthally varying polarized vector field. By using the optical tweezers, we demonstrate the existence of arbitrarily tunable OAM. The arbitrarily tunable OAM we presented has the same characteristics as the well-defined integer OAM: intrinsic OAM, uniform local OAM and intensity ring, and propagation stability. The arbitrarily tunable OAM has unique natures: it is allowed to be flexibly tailored and the radius of the focusing ring can have various choices for a desired OAM.

## Results

### Theory

We have predicted that a vector field with the vector potential of 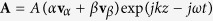 is able to carry the two parts of OAM flux associated with the azimuthal gradient^29^


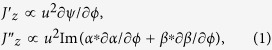


where *A* is the complex amplitude of **A**, as *A* = *u*exp(*jψ*) by its module *u* and its phase *ψ*. 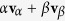 is a unit vector describing the distribution of polarization state of **A**, with 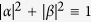. The unit vectors **v**_*α*_ and **v**_*β*_ indicate a pair of orthogonal polarization states and can be represented by a pair of antipodal points on the Poincaré sphere^28^,^30^. If *α* and *β* are the functions of the transverse coordinates (*r*, *ϕ*), **A** is a vector field; otherwise **A** degenerates into a scalar field. For the light field **A**, the OAM per photon can be identified as


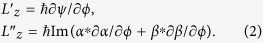


In fact, 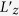 is the well-defined OAM carried by the scalar vortex field with the helical phase of exp(*jmϕ*), with an intrinsic and eigen OAM of *mħ* per photon^1^. We call 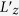 as the photon OAM of the first kind. 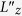 is associated with the vector field. We have demonstrated the OAM from the curl of polarization, called as the photon OAM of the second kind, which can be carried by the radially varying hybridly polarized vector fields only^29^. Since 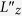 is always zero for a scalar field because *α* and *β* are independent of *ϕ*, the vector field should be a unique opportunity for tailoring of 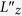. With Eq. (2), the nonzero 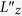 requires the polarization states to be azimuthally varying. Although the local linearly polarized vector fields^27^ and the hybridly polarized vector fields^28^ exhibit both the azimuthally varying polarization states, 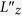 is still null (Supplementary S1).

**Figure 1:**
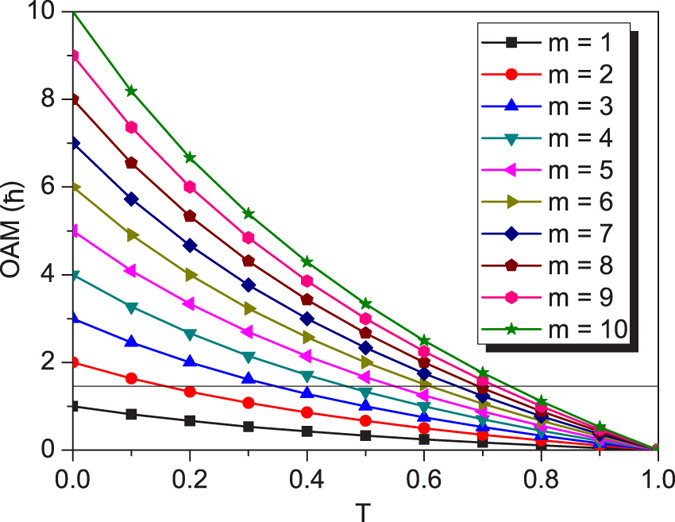
Fractional OAM carried by the azimuthally varying polarized vector fields. Dependence of the OAM on the topological charge *m* and the relative intensity fraction *T*. The black thin horizontal line indicates a certain given OAM (or *m*_*eff*_) and has a series of intersections with the color curves, in which each intersection represents a combination of *m* and *T* for achieving of that given OAM.

Let us refocus on the two azimuthally variant polarized vector fields^27^,^28^ again, where a pair of orthogonally polarized components with the completely opposite helical phases of 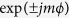 have the *equal* intensity. This brings us an inspiration that the most possible solution for the nonzero 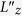 may be break through the balance in intensity between the two orthogonal components. In such a situation, the unit vector representing the distribution of polarization states should be rewritten as


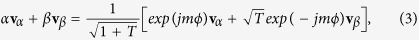


where *T* is the relative intensity fraction between the two orthogonal components within a range of 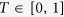. With Eq. (2), we easily have 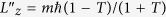, where we define 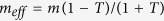 and the OAM per photon is *m*_*eff*_*ħ*. Clearly, *T* as a degree of freedom can be used to continuously tailor the OAM within a range of [0, *mħ*], although *m* can only take an integer (Fig. 1). It is very interesting and surprising that for a desired OAM or *m*_*eff*_, it can be achieved by a variety of combinations of *m* and *T*, as a series of intersections of the color curves with the thin horizontal line (Fig. 1). In the extreme case of *T* = 1, it has been confirmed 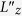 = 0 (Supplementary S1). In the extreme case of *T* = 0, the vector field described in Eq. (3) degenerates into a scalar vortex field with the helical phase of exp(*jmϕ*), carrying the OAM of *mħ* per photon^1^. Obviously, 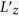 should belong to a special case of 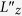 when *T* = 0. In particular, the phase exp(*jψ*) can be in fact incorporated into *α* and *β*, and 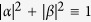 is still held. Therefore, 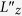 should be a *general* form of the OAM associated with the azimuthal gradient, and can be called as the *general* OAM of the first kind and is written as 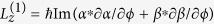.

### Experiment

**Figure 2:**
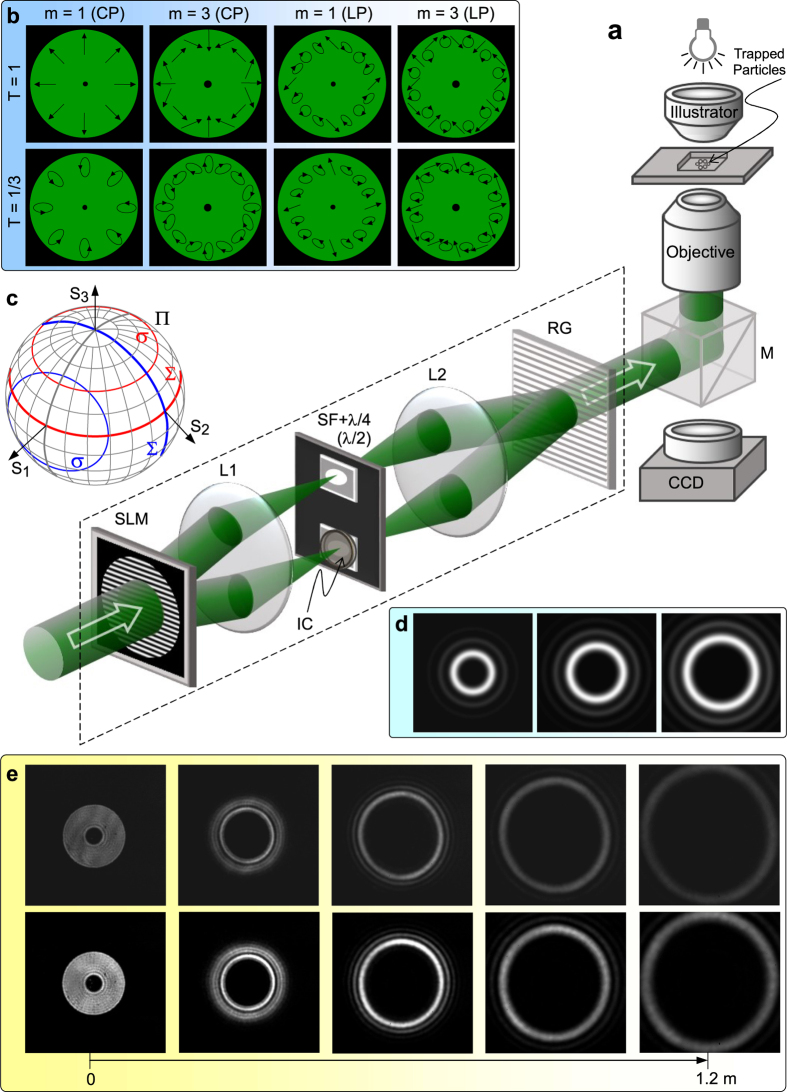
Experimental configuration to validate the fractional OAM we predicted. (**a**) Optical tweezers system, the dashed-line parallelogram shows the generation unit of azimuthally varying vector fields. (**b**) Diagrammatic drawings of the distribution of the polarization state for the azimuthally varying vector fields, the 1^st^ and 2^nd^ (3^rd^ and 4^th^) columns show the vector fields created by a pair of right- and left-handed CP (*x* and *y* LP) bases, the 1^st^ and 2^nd^ rows are the cases of the relative intensity fraction, *T* = 1 and *T* = 1/3, respectively. (**c**) Geometric presentation of the Poincaré sphere for the polarization states of the azimuthally varying vector fields shown in (**b**). (**d**) Simulated multi-ring structures of the focusing fields of the azimuthally varying top-hat vector fields with *m* = 10, 14 and 18 when *T* = 1/3. (**e**) Propagation evolutions of the azimuthally varying vector fields with the top-hat profile in free space, shown by the experimentally measured intensity profiles at a series of distances from the output plane. The measured intensity pattern of the scalar vortex top-hat field with the helical phases of exp(+*j*20*ϕ*) undergoes an evolution from the top-hat profile at *z* = 0 to the multi-ring structure at *z* = 1.2 m (top row). For the vector field created by a pair of orthogonal polarized base fields with the helical phases of exp(±*j*20*ϕ*) when *T* = 0.32, its propagation behavior (bottom row) has no difference from the scalar vortex field (top row), implying that the vector field is propagation stable.

To confirm the feasibility of the arbitrarily tunable OAM we presented, the focused vector field as the optical tweezers is a useful tool (Fig. 2a). The generation unit of the vector fields is very similar to that used in refs 24 and 25, but has a unique difference that the ±1st orders carrying the completely opposite helical phases of exp(±*jmϕ*) can have the different intensity (Methods). Thus the demanded vector field can be written as


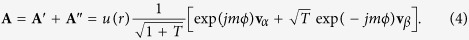


where *u*(*r*) has the top-hat profile with *u*(*r*) = *U*_0_circ(*r*/*R*_0_) (Fig. 2b). *U*_0_ is constant amplitude, and circ(*r*/*R*_0_) is a well-known circular function defined as circ(*r*/*R*_0_) = 1 within *r* < *R*_0_ but circ(*r*/*R*_0_) = 0 within *r *> *R*_0_, where *R*_0_ is the field radius (Methods).

As examples, Fig. 2b shows the schematic sketches of the polarization states of the azimuthally variant polarized vector fields with *m* = 1 and 3 as well as *T* = 1 and 1/3. For the vector fields created by a pair of orthogonal circularly polarized (CP) spinors [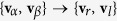 in Eq. (4)], shown in the first column with *m* = 1 (second column with *m* = 3) of Fig. 2b, its polarization state exhibits the azimuthally variant local linear polarizations when *T* = 1, which traverse once (thrice) all points located at the equator on the Poincaré sphere Π (ref. 30, Supplementary S2 and Fig. S1) with the Stocks parameter of *S*_3_ = 0 shown by the thick red curve in Fig. 2c; whereas the polarization states exhibit the azimuthally variant orientation of elliptical polarizations with the same ellipticity when *T* = 1/3, which traverse once (thrice) all points located at the north-latitude 30° circle (*S*_3_ = 1/2) on Π, shown by the thin red curve in Fig. 2c. In contrast, for the vector fields created by a pair of orthogonal linearly polarized (LP) bases [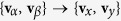 in Eq. (4)] in the third (fourth) column of Fig. 2b, the polarization states undergo the azimuthal variation from the linear, through elliptic to circular polarizations when *T* = 1, which traverse once (thrice) all points located at the great circle (*S*_1_ = 0) on Π for *m* = 1 (*m* = 3), shown by the thick blue curve in Fig. 2c; while the polarization states undergo the azimuthal variation from the linear to elliptic polarizations but does not occur the circular polarization when *T* = 1/3, which traverse once (thrice) all points located at the *S*_1_ = 1/2 circle on Π for *m* = 1 (*m* = 3), shown by the thin blue curve in Fig. 2c. For a more general case (Supplementary S2 and Fig. S2), a pair of orthogonally polarized bases 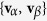 in Eq. (4) correspond to any pair of antipodal points on Π. Thus the polarization states of the created vector field are described by all points located at a circle *σ* on Π. The circle *σ* is the intersection of Π with the plane *σ* normal to the connecting line between the antipodal points. The plane *σ* has a distance of 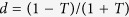 from the center of Π. We further define a great circle ∑, which is the intersection of Π with a plane passing the center of Π and being parallel to the plane *σ*. In fact, the Poincaré sphere can also be used to characterize the arbitrarily tunable OAM, which is equal to the distance *d* of the plane *σ* from the center of Π, in units of *mħ*. Of course, the OAM can also be characterized as 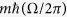, by a solid angle Ω subtended by the spherical zone sandwiched between the two circles *σ* and ∑ on Π, with 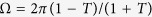 (Supplementary S2). In particular, we should emphasize that the arbitrarily tunable OAM is independent of the choice of spinors.

It is of great importance to explore the propagation stability of the vector fields carrying the arbitrarily tunable OAM. The measured intensity pattern of the scalar vortex top-hat field with the helical phases of exp(+*j*20*ϕ*) undergoes an evolution from the top-hat profile at *z* = 0 to the multi-ring structure at *z* = 1.2 m (top row in Fig. 2e). For the vector field created by a pair of orthogonal polarized bases with the opposite helical phases of exp(±*j*20*ϕ*) when *T* = 0.32, its propagation behavior has no difference from the scalar vortex top-hat field, implying that the vector field is propagation stable (bottom row in Fig. 2e) and the arbitrarily tunable OAM we presented is always remained at any plane during the propagation. For the vector field created by a pair of orthogonal polarized bases with the helical phases of exp(+*j*20*ϕ*) and exp(−*j*5*ϕ*) when *T* = 0.32, this vector field is unstable during its propagation (Supplementary S3 and Fig. S3), resulting in the spatial separation of different OAM states. Therefore, the fractional OAM cannot be remained at any plane during the propagation.

**Figure 3:**
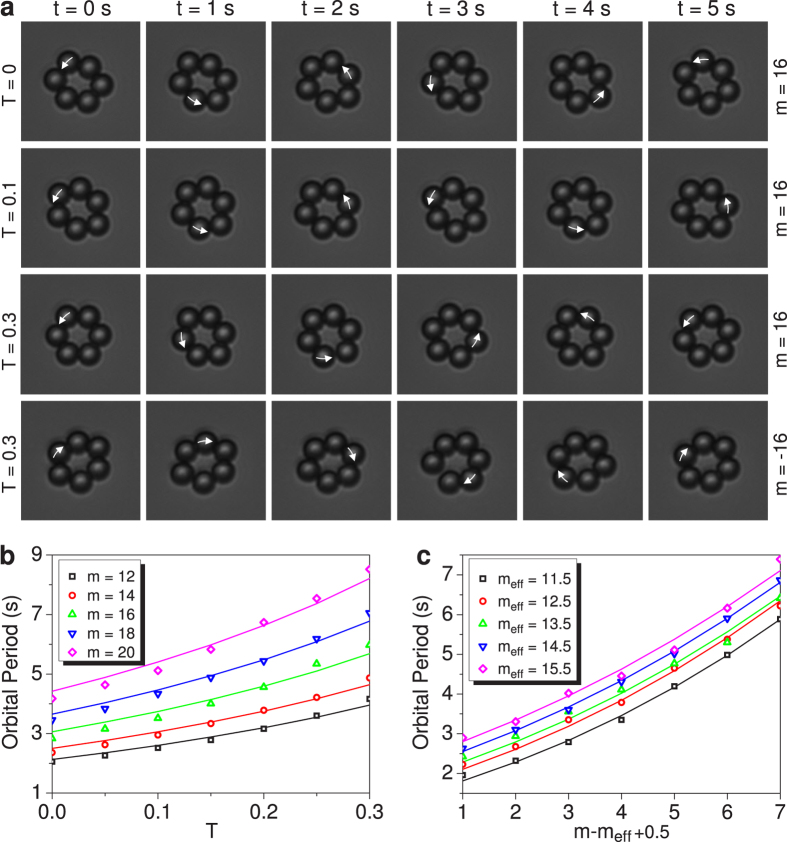
Observed orbital motion of trapped particles around the ring focus produced by azimuthally varying vector fields. **(a)** Snapshots of orbital motion of the trapped particles around the ring focus generated by the azimuthally varying vector fields (Movie). **(b)** Dependence of the period *τ* of the orbital motion of the trapped particles on *T* for five different *m*. The symbols are the measured periods and the corresponding curves are the fitting results obtained by 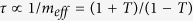 (Supplementary S4). **(c)** Dependence of the period *τ* of the orbital motion of the trapped particles on *m* for five different *m*_*eff*_. The symbols are the measured periods and the corresponding curves are the fitting results obtained by 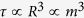 because *R* is approximately in direct proportion to *m* (Supplementary S4).

The created azimuthally variant polarized vector field is introduced into the optical tweezers system (Method), which is a very useful tool to explore the photon OAM by observing and recording the orbital motion of the trapped particles (Video). We intercept the time-lapse photos of the orbital motion of the trapped particles (Fig. 3a). For the vector field with *m* = 16 and *T* = 0 (the vector field degenerates into a scalar vortex field with *m* = 16), the trapped particles move around the principal ring focus with an orbital period of *τ* ~ 2.47 s (in first row). When *T* is changed to *T* = 0.1, the orbital period of the trapped particles increases to *τ* ~ 2.94 s (in second row). When *T* is further increased to *T* = 0.3, the orbital period further increases to *τ* ~ 4.75 s (in third row). When *m* is switched from *m* = 16 to *m* = −16 when keeping *T* = 0.3, the motion direction of the trapped particles is synchronously reversed with an orbital period of *τ* ~ 4.99 s (in fourth row) (Video). The slight difference of the periods is due to the slight difference of the intensity and shape of the two bases, of course, the activity of the particle and water has also the influence. However, for the vector fields with *T* = 1 (the hybridly polarized vector fields reported in ref. 28), no orbital motion of the trapped particles is observed, implying that such a kind of vector fields carry no OAM. The dependences of the orbital period *τ* of the trapped particles, on *T* for different *m* (Fig. 3b) and on *m* for different effective topological charge *m*_*eff*_ (Fig. 3c), indicate that the measured orbital periods (symbols) are in good agreement with the fitted curves by the respective formulae 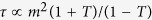 and 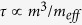 (Supplementary S4). The observed results clarify the following facts. (i) The azimuthally varying polarized vector fields could indeed carry the arbitrary OAM and (ii) the choice of orthogonally polarized bases has no influence on the arbitrary OAM.

## Discussion

### Damping model

**Figure 4:**
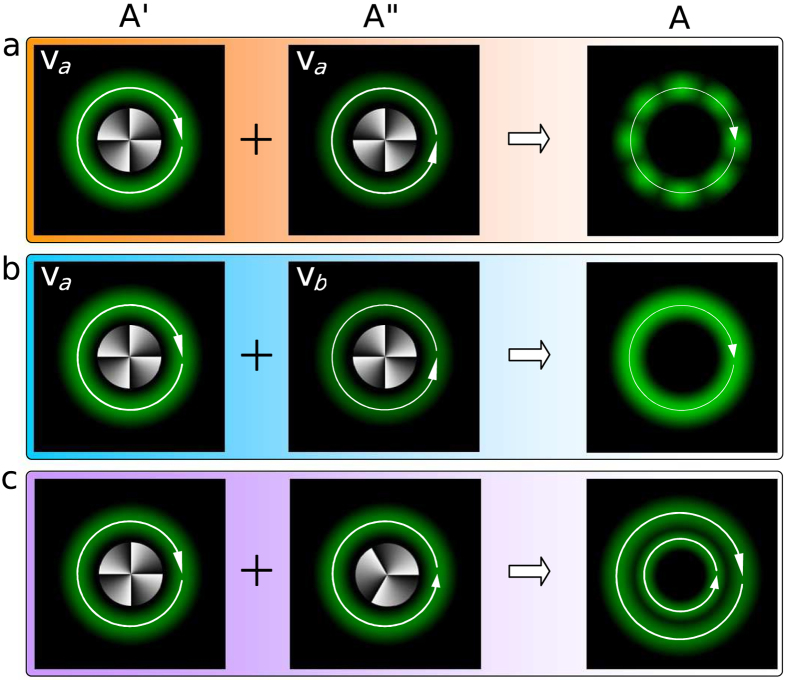
Damping model for intuitively understanding of the fractional OAM. (**a**) 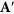 and 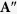 have the same polarization and carry the opposite OAMs of ±*mħ* per photon, 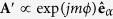 and 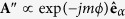, the total field 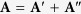 has the nonuniform annular ring and may carry the local nonuniform OAM. (**b**) 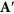 and 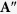 have the orthogonal polarizations to each other and carry the opposite OAMs of ±*mħ* per photon, 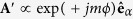 and 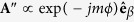, the total field 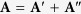 has the uniform annular ring and may carry the arbitrary and local uniform OAM. (**c**) 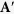 and 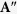 carry the OAMs of +*mħ* and −*m'ħ* per photon, 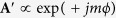 and 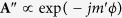, the total field 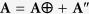 is unstable during its propagation because 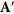 and 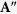 will separate in the radial dimension, which is independent of polarization states of 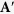 and 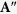. Green patterns are schematic diagrams of the intensity distribution, Gray patterns in center of any picture are schematic diagrams of the vortex phase, the circles with the arrow show the direction of OAM and the lengths of arrow show the magnitudes of OAM.

Although the above theoretical and experimental results have unveiled and demonstrated the realization of fractional OAM by using the vector fields, we now provide a very simple and intuitive physical model—*damping model*—for understanding the fractional OAM (Fig. 4). For the orbital motion of a classical particle, if the damping is introduced, its motion speed will become slow and then its OAM will also become small synchronously. This classical damping model enlightens us “Whether introducing the suitable *damping* is able to flexibly realize the control of photon OAM?” A scalar vortex field 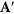 with the helical phase of exp(+*jmϕ*) and the polarization state **v***α* is able to drive the orbital motion of the trapped particle due to the presence of angular momentum flux. Of course, another scalar vortex field 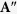 with the *opposite* helical phase of exp(−*jmϕ*) will provide the *opposite*-sense angular momentum flux, as a damping for 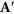. If 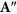 has the same polarization state as 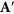, thus the total field 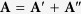 is still a scalar field with the polarization state **v***α*. The angular momentum flux provided by 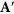 can be completely or partially canceled by the damping field 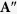, which is able to realize the continuously tunable net angular momentum flux and then the arbitrary OAM (Fig. 4a). However, this is not what we ideally expected, because the interference between 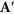 and 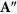 gives rise to the nonuniformity in both the ring intensity and the local OAM (Fig. 4a). Fortunately, the vector nature of photons may be provide a solution. If the polarization state of 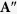, described by the unit vector **v**_*β*_, is orthogonal to **v***α* of 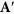, the total field 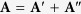 becomes into a *vector* field^26^,^27^,^28^. Its net OAM should also be continuously tunable (Fig. 4b). Moreover, it is of extreme importance that the intensity ring and the local OAM are both uniform in the azimuthal dimension (Fig. 4b). There is still a question why the topological charges of 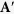 and 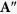 are selected to be completely opposite in the above. Based on the *damping* model, it seems to be in principle allowed that 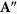 has a helical phase of exp(−*jm*′*ϕ*) with 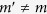. However, such a choice is in fact unsuitable because the total field **A** is unstable during its propagation (Fig. 4c), because two fields carrying the helical phases with the different topological charges can never always overlap in space.

### Quantum understanding

We always attempt to understand the fractional OAM from the quantum point view. As is well known, the Hamiltonian 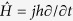 and the *z* component of OAM 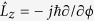 are two commuting operators, i.e. 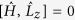, so both have the common eigen wave function. The light field described by Eq. (4) is composed of two orthogonally polarized components, which can be rewritten as 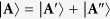. We can easily confirm that 
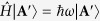
 and 
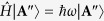
, and 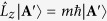 and 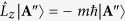. Clearly, 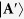 (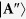) is indeed the common wave function of 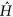 and 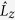, with the respective eigen values of 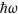 and 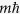 (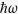 and 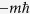). Thus the photon states described by 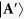 and 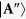 are a pair of orthogonal polarized eigen wave functions of OAMs and have the eigen OAMs of 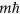 and 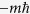 per photon, respectively. Equation (4) in the manuscript also describes in fact a mixing wave function composed of two eigen OAM states (with the eigen OAMs of 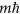 and 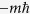 per photon). In other words, the photon is in a mixing OAM state composed of two eigen OAM states. We can easily obtain that the photon in this mixing state has an expectation value of OAM as 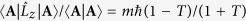 per photon.

We have presented a solution of the photon OAM for long-time challenge. The photon OAM we have proved has novel and unique natures: (i) it is continuously tunable within a range of [−*mħ*, *mħ*], (ii) it has the uniformity, and (iii) the light field carrying the arbitrarily tunable OAM has the uniform intensity ring and has the propagation stability. We presented the general OAM of the first kind, associated with the azimuthal gradient, which extends the OAM carried by the scalar vortex fields to the OAM carried by the azimuthally varying polarized vector fields. We have also extended the Poincaré sphere to represent the arbitrarily tunable OAM. Our idea may spur further independent insights into the generation of natural waves carrying the arbitrarily tunable OAM. The current technology trend has been perceived to direct from fundamental investigations towards probing its viabilities for surprising applications. The fast-moving exploitation on such diverse areas has pushed for further development on OAM generation technology.

## Methods

### Creation of azimuthally varying polarized vector fields

We follow the method similar to those used in refs 27 and 28 for creating the demanded azimuthally varying polarized vector fields (the part enclosed by the dashed-line box in Fig. 2a). The used light source is a continuous-wave laser operating at a wavelength of 532 nm (Verdi-5, Coherent Inc.), which outputs a near fundamental Gaussian mode. The laser beam is expended and then the collimated beam illuminates the computer-generated holographic grating displayed at the spatial light modulator (SLM), located at the input plane of the 4f system composed of a pair of lenses (L1 and L2). The incoming beam is diffracted by the computer-generated holographic grating with the amplitude transmittance of *t*(*x*, *y*) = [1 + *γ*cos(2*πf*_0_*x *+ *δ*)]/2 with the additional azimuthally varying phase *δ* = *mϕ*, where *ϕ* is the azimuthal angle and *m* is the topological charge. The diffracted ±1st orders are selected by a spatial filter (SF) located at the spatial frequency plane of the 4f system. The ±1^st^ orders are transferred by a pair of 1/4 (or 1/2) wave plates into a pair of orthogonal circularly (or linearly) polarized bases. In particular, an intensity controller (IC) composed of a linear polarizer and a 1/2 wave plate is inserted into the −1^st^ order path to achieve the continuous change of the relative intensity fraction *T* between the two paths. Finally, the orthogonal circularly (or linearly) polarized ±1^st^ orders are recombined by a Ronchi grating (RG) placed at the output plane of the 4f system to create the demanded azimuthally varying polarized vector fields, as shown in Eq. (4). Thus we can select the different topological charge *m*, the different relative intensity fraction *T* and the different orthogonally polarized bases 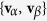, to create various azimuthally variant polarized vector fields.

### Optical tweezers and the indirect measurement of OAM

The direct method to measure the topological charge is mainly associated with directly detecting the phase distribution of the light field, such as detecting the interference patterns. This arbitrarily tunable OAM we proposed here is not associated directly with the vortex phases with the fractional topological charge, so we cannot directly measure the arbitrarily tunable OAM based on the measurement of fractional topological charge. We use the indirect method to measure the arbitrarily tunable OAM and confirm our idea by the optical tweezers.

The created azimuthally varying polarized vector field is introduced into an optical tweezers system composed of an inverted microscope including a 60× objective with NA = 0.75 (Fig. 2a). The neutral isotropic colloidal microspheres with the almost same diameter of 2.8 μm are dispersed in a layer of sodium dodecyl sulfate solution between a glass coverslip and a microscope slide. The azimuthally varying vector field with the top-hat profile is focused into a multi-ring structure composed of a principal ring and secondary rings (Fig. 2d), and laser power in the focal region is kept at ~15 mW. The neutral microparticles can be trapped in the principal ring. The motion behavior of the trapped particles can indirectly characterize the photon OAM carried by the light field. If the trapped isotropic particles in the ring optical tweezers move around the ring orbit, implying that the azimuthally variant vector fields will have the capability to exert torque to the trapped isotropic particles. No doubt this verifies the presence of photon OAM. The motion direction and speed of the trapped particles indicate the sense and magnitude of the photon OAM carried by the azimuthally variant vector field. If no motion of the trapped isotropic particles is observed around the ring, implying that the fields carry no photon OAM. Through taking the video of the motion of the trapped particles, the orbital period or motion speed of the trapped particles can be measured, which indirectly characterize the OAM.

## Additional Information

**How to cite this article**: Pan, Y. *et al*. Arbitrarily tunable orbital angular momentum of photons. *Sci. Rep.* **6**, 29212; doi: 10.1038/srep29212 (2016).

## Supplementary Material

Supplementary Information

Supplementary Video
