# Supplementary material for: Arbitrarily tunable orbital angular momentum of photons
Source: Sci Rep. 2016 Jul 5;6:29212. doi: 10.1038/srep29212 (PMC4932607; doi:10.1038/srep29212)
Supplement: Supplementary Information [file srep29212-s1.doc]

Supplementary Materials for

**Arbitrarily tunable Orbital Angular Momentum of Photons**

Yue Pan, Xu-Zhen Gao, Zhi-Cheng Ren, Xi-Lin Wang, Chenghou Tu, Yongnan Li, Hui-Tian Wang*

**S1. The OAM carried by azimuthally varying polarized vector fields**

The created azimuthally vector fields by a pair of any orthogonal polarized base fields with the equal intensity (*T* = 1) and the completely opposite spiral phases. Thus the created vector field can be rewritten as

$\text{A}\propto\alpha\text{v}_{\alpha}+\beta\text{v}_{\beta}=\frac{1}{\sqrt{2}}\left[\exp\left(jm\varphi\right)\text{v}_{\alpha}+\exp\left(-jm\varphi\right)\text{v}_{\beta}\right]$.

When a pair of orthogonal polarized base vectors is orthogonal right- and left-handed circular polarizations $(\text{v}_{\alpha},\text{v}_{\beta})\to(\text{v}_{r},\text{v}_{l})$ in ref. 24 or orthogonal linear polarizations $(\text{v}_{\alpha},\text{v}_{\beta})\to(\text{v}_{x},\text{v}_{y})$ in Ref. 25, the created vector field is the azimuthally varying local linearly polarized vector field in Ref. 24 or and the azimuthally varying hybridly polarized vector fields in Ref. 25.

We have and . We can give as follows

$$\begin{matrix} \alpha\partial\alpha^{*}/\partial\varphi=\frac{1}{\sqrt{2}}\exp(jm\varphi)\partial\left[\frac{1}{\sqrt{2}}\exp(-jm\varphi)\right]/\partial\varphi=-\frac{1}{2}jm \\ \beta\partial\beta^{*}/\partial\varphi=\frac{1}{\sqrt{2}}\exp(-jm\varphi)\partial\left[\frac{1}{\sqrt{2}}\exp(jm\varphi)\right]/\partial\varphi=\frac{1}{2}jm \end{matrix}$$

And then

$$\begin{matrix} \left(\alpha\partial\alpha^{*}/\partial\varphi-\alpha^{*}\partial\alpha/\partial\varphi\right)+\left(\beta\partial\beta^{*}/\partial\varphi-\beta^{*}\partial\beta/\partial\varphi\right) \\ =\left[-\frac{1}{2}jm-\left(-\frac{1}{2}jm\right)^{*}\right]+\left[\frac{1}{2}jm-\left(\frac{1}{2}jm\right)^{*}\right]=0 \end{matrix}$$

With Eq. (1), we have

$$J_{z}^{(2)}=0$$

and then

$$L_{z}^{(2)}=0$$

As a result, if only a pair of orthogonal polarized base fields have the equal intensity, the created vector fields carry no OAM ($L_{z}^{(2)}=0$), which is independent of the choice of a pair of orthogonal polarized base fields.

**S2. Poincaré Sphere, Distributions of polarization states, and OAM**

**Poincaré Sphere.** Any polarization state can be described by a pair of orthogonal right- and left-handed circularly polarized (CP) bases, **v***_r_* and **v***_l_*, as

$\hat{\text{P}}=a_{r}\text{v}_{r}+a_{l}\text{v}_{l}$ with $|a_{r}|^{2}+|a_{l}|^{2}=1$. (S1)

To describe all possible polarization states of a polarized field, Poincaré sphere is a simple and convenient geometric representation (Ref. 30 and Fig. S1).


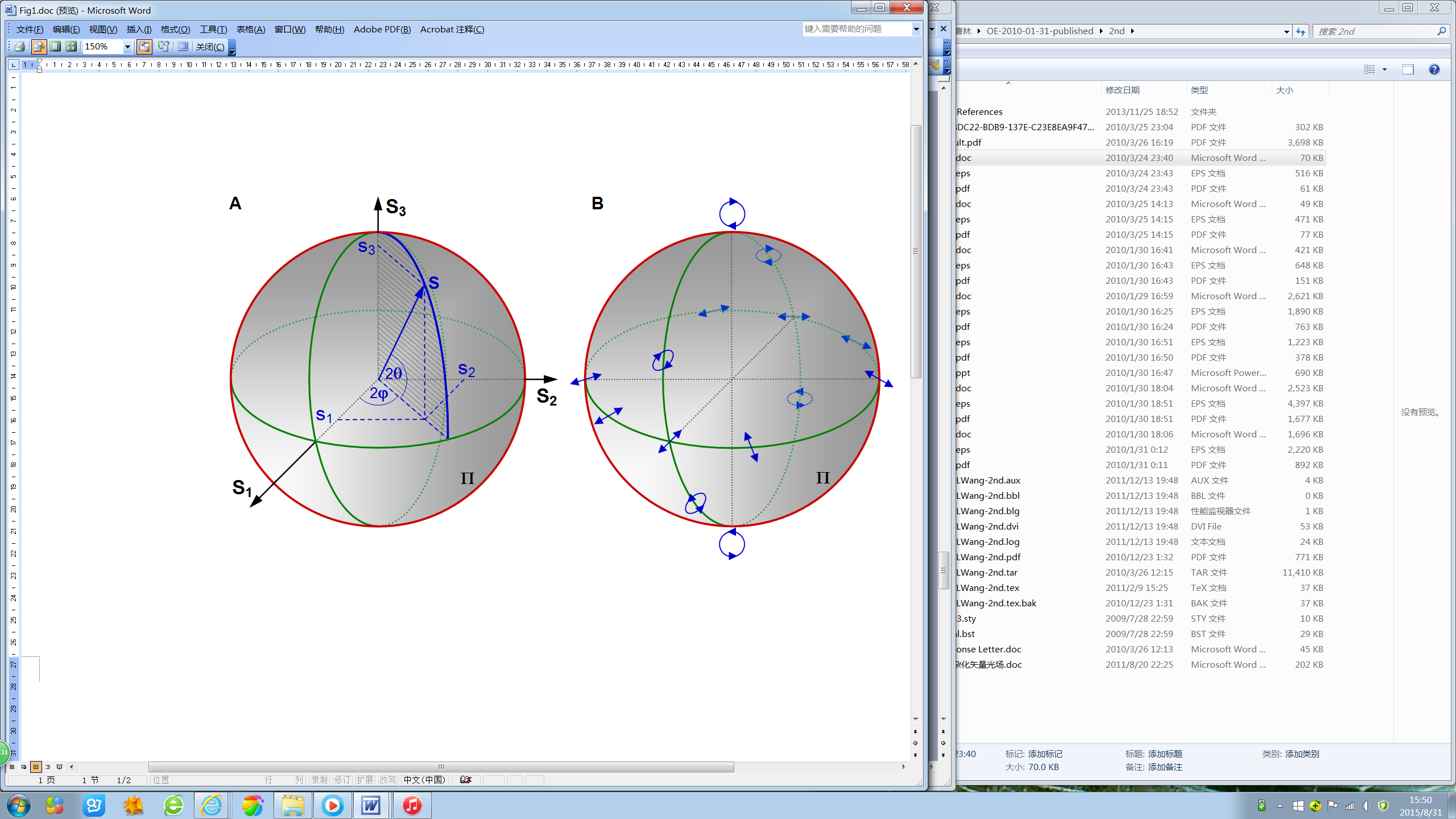


Fig. S1 Poincare sphere.

**a**

**b**

In mathematical linguistics, the polarization state of a polarized light can also be described by a 3×1 “Jones vector”, three normalized Stokes parameters, *S*_1_, *S*_2_ and *S*_3_, have the following connection with *a_r_* and *a_l_* as follows

$\left[\begin{matrix} S_{1} \\ S_{2} \\ S_{3} \end{matrix}\right]=\left[\begin{matrix} 2\mathrm{Re}(a_{r}^{*}a_{l}) \\ 2\mathrm{Im}(a_{r}^{*}a_{l}) \\ |a_{r}|^{2}-|a_{l}|^{2} \end{matrix}\right]$ with $S_{1}^{2}+S_{2}^{2}+S_{3}^{2}=1$. (S2)

The three Stocks parameters, *S*_1_, *S*_2_ and *S*_3_, can be considered as sphere’s Cartesian coordinates of a point on a unit sphere to construct the Poincaré sphere Π. Of course, the Poincaré sphere Π with a unit radius can also be defined by the latitude angle 2*θ* and the longitude angle 2*ϕ* in spherical coordinate system. *S*_1_, *S*_2_ and *S*_3_ can be represented in terms of 2*θ* and 2*ϕ*, as follows

$\left[\begin{matrix} S_{1} \\ S_{2} \\ S_{3} \end{matrix}\right]=\left[\begin{matrix} \cos2\theta\cos2\phi\\ \cos2\theta\sin2\phi\\ \sin2\theta\end{matrix}\right]$. (S3)

The factor 2 in the front of 2*θ* and 2*ϕ* obeys the 2→1 homeomorphism between the SU(2) space of the Jones calculus and the topological SO(3) space of the Poincaré sphere Π, and ensures that one point on Π corresponds to a unique polarization state and vice versa. In fact, 2*ϕ* (0 ≤ 2*ϕ* < 2*π*) specifies the orientation of the polarization ellipse and 2*θ* (−*π*/2 ≤ 2*θ* ≤*π*/2) characterizes the ellipticity and the sense of the polarization ellipse. Since 2*θ* is positive (negative) according as the polarization is right-handed (left-handed), the right-handed (left-handed) polarization is represented by points on Π which lie the northern (southern) hemisphere of Π, while linear polarization is represented by points on the equator. The right-handed (left-handed) circular polarization is represented by the north (south) pole.

**Distributions of polarization states.** By using the method as mentioned above, the created vector field is given by Eq. (4), in a pair of orthogonal polarized bases $\{\text{v}_{\alpha},\text{v}_{\beta}\}$, which are allowed to correspond to any pair of antipodal points on Π (Ref. 30).

If the orthogonal polarized bases are selected to be the orthogonal CP spinors, as $\{\text{v}_{\alpha},\text{v}_{\beta}\}\to\{\text{v}_{r},\text{v}_{l}\}$, the polarization state of the created vector field given in Eq. (4) should be

$\hat{\text{P}}=\alpha\text{v}_{\alpha}+\beta\text{v}_{\beta}=\frac{1}{\sqrt{1+T}}\exp(jm\varphi)\text{v}_{r}+\frac{\sqrt{T}}{\sqrt{1+T}}\exp(-jm\varphi)\text{v}_{l}$. (S4)

When comparing Eq. (S4) with Eq. (S1), we obtain from Eq. (S2)

$\left[\begin{matrix} S_{1} \\ S_{2} \\ S_{3} \end{matrix}\right]=\left[\begin{matrix} 2\cos(2m\varphi)\sqrt{T}/(1+T) \\ -2\sin(2m\varphi)\sqrt{T}/(1+T) \\ (1-T)/(1+T) \end{matrix}\right]$. (S5)

Clearly, the polarization states of the vector fields created by a pair of orthogonal CP spinors locate at the circle $S_{3}=(1-T)/(1+T)$ on Π.

If the orthogonal polarized bases are selected to be the orthogonal LP bases, as $\{\text{v}_{\alpha},\text{v}_{\beta}\}\to\{\text{v}_{x},\text{v}_{y}\}$, the polarization state of the created vector field given in Eq. (4) should be

$\hat{\text{P}}=\alpha\text{v}_{\alpha}+\beta\text{v}_{\beta}=\frac{1}{\sqrt{1+T}}\exp(jm\varphi)\text{v}_{x}+\frac{\sqrt{T}}{\sqrt{1+T}}\exp(-jm\varphi)\text{v}_{y}$. (S6)

With the aid of $\text{v}_{x}=(\text{v}_{r}+\text{v}_{l})/\sqrt{2}$ and $\text{v}_{y}=-j(\text{v}_{r}-\text{v}_{l})/\sqrt{2}$, and with Eq. (S2), we can obtain the Stocks parameters of the polarization state described by Eq. (S7)

$\left[\begin{matrix} S_{1} \\ S_{2} \\ S_{3} \end{matrix}\right]=\left[\begin{matrix} (1-T)/(1+T) \\ 2\cos(2m\varphi)\sqrt{T}/(1+T) \\ -2\sin(2m\varphi)\sqrt{T}/(1+T) \end{matrix}\right]$. (S7)

Clearly, the polarization states of the vector fields created by a pair of orthogonal LP bases locate at the circle $S_{1}=(1-T)/(1+T)$ on Π.

The created vector fields with *m* = 1 and 3 as well as *T* = 1 and 1/3 exhibit the azimuthally varying polarization states dependent on both *m* and *T* [Fig. 3(c)]. For the vector fields created by a pair of orthogonal CP spinors [$\{\text{v}_{\alpha},\text{v}_{\beta}\}\to\{\text{v}_{r},\text{v}_{l}\}$ in Eq. (4) and Eq. (S4)] in the 1st (2nd) column of Fig. 3(b), its polarization states exhibit the azimuthally varying linear polarizations when *T* = 1, which traverse once (thrice) all points located at the equator on Π  with *S*_3_ = 0 for *m* = 1 (*m* = 3), shown by the thick red curve in Fig. 3(c); whereas the polarization states exhibit the azimuthally varying orientation of elliptical polarizations with the same ellipticity when *T* = 1/3, which traverse once (thrice) all points located at the north-latitude 30º circle (*S*_3_ = 1/2) on Π for *m* =1 (*m* = 3), shown by the thin red curve in Fig. 3(c).

In contrast, for the vector fields created by a pair of orthogonal linearly polarized (LP) bases [$\{\text{v}_{\alpha},\text{v}_{\beta}\}\to\{\text{v}_{x},\text{v}_{y}\}$ in Eq. (4) and Eq. (S6)] in the 3rd (4th) column of Fig. 3(b), the polarization states undergo the azimuthal variation from the linear, through elliptic to circular polarizations when *T* = 1, which traverse once (thrice) all points located at the great circle (*S*_1_ = 0) on Π for *m* =1 (*m* = 3), shown by the thick blue curve in Fig. 3(c); while the polarization states undergo the azimuthal change from the linear to polarizations but does not occur the circular polarization when *T* = 1/3, which traverse once (thrice) all points located at the *S*_1_ = 1/2 circle on Π for *m* = 1 (*m* = 3), shown by the thin blue curve in Fig. 3(c).

We can extend to a general case that the azimuthally varying polarized vector field is created by a pair of orthogonally polarized bases $\{\text{v}_{\alpha},\text{v}_{\beta}\}$, which correspond to any pair of antipodal points on the Poincaré sphere Π. For instance, as shown in Figs. S2a and S2b, a pair of orthogonally polarized bases $\{\text{v}_{\alpha},\text{v}_{\beta}\}$ are elliptical polarizations orthogonal to each other. The polarization states are described by all points located at a circle *σ* on Π. The circle *σ* is the intersection of Π with the plane *σ* normal to the connecting line between the antipodal points. The plane *σ* has a distance of $d=(1-T)/(1+T)$ from the center of Π. We further define a great circle Σ, which is the intersection of Π with a plane passing through the center of Π and being parallel to the plane *σ*. For the two special cases of $\{\text{v}_{\alpha},\text{v}_{\beta}\}\to\{\text{v}_{r},\text{v}_{l}\}$ and $\{\text{v}_{\alpha},\text{v}_{\beta}\}\to\{\text{v}_{x},\text{v}_{y}\}$ [Fig. 3(c), Figs. S2c and S2d], the two circles *σ* correspond to the circles $S_{3}=(1-T)/(1+T)$ and $S_{1}=(1-T)/(1+T)$, respectively; the two great circles Σ are the great circles *S*_3_ = 0 and *S*_1_ = 0, respectively.

**OAM.** In fact, the Poincaré sphere can also be used to characterize the arbitrarily tunable OAM carried by the azimuthally varying polarized vector field, that is to say, the photon OAM is equal to a distance $d=(1-T)/(1+T)$ of the plane *σ* from the plane Σ, in units of *mħ*. Of course, the photon OAM can also be characterized as by a solid angle Ω subtended by the spherical zone [yellow areas in Figs. S2a and S2b] sandwiched between the two circles *σ* and Σ on Π, with $\text{Ω}=2\pi(1-T)/(1+T)$. We discuss the two special cases as mentioned above, as shown in Figs. S2c and S2d, which also correspond to Fig. 3(c) in the main text. For the special case of $(\text{v}_{\alpha},\text{v}_{\beta})\to(\text{v}_{r},\text{v}_{l})$ (Fig. S2c), a pair of orthogonally polarized base fields are right- and left-handed circular polarizations corresponding to the north and south poles on Π, the great circle Σ is the equator with *S*_3_ = 0 and the circle *σ* correspond to the circle of $S_{3}=(1-T)/(1+T)$. The photon OAM is equal to *S*_3_ in units of *mħ*, or can be represented as by a solid angle Ω subtended by the spherical zone (red area in Fig. S2c) sandwiched between the two circles *σ* and Σ on Π, with $\text{Ω}=2\pi(1-T)/(1+T)=2\pi S_{3}$. For the special case of $(\text{v}_{\alpha},\text{v}_{\beta})\to(\text{v}_{x},\text{v}_{y})$ (Fig. S2d), a pair of orthogonally polarized base fields are *x*- and *y*-linear polarizations corresponding to a pair of points with *S*_1_ = 1 and *S*_1_ = −1 in the equator on Π, the great circle Σ is the circle with *S*_1_ = 0 and the circle *σ* correspond to the circle of $S_{1}=(1-T)/(1+T)$. The photon OAM is equal to *S*_1_ in units of *mħ*, or can be represented as by a solid angle Ω subtended by the spherical zone (blue area in Figs. S2d) sandwiched between the two circles *σ* and Σ on Π, with $\text{Ω}=2\pi(1-T)/(1+T)=2\pi S_{1}$.

S_1_

S_2_

S_3_

**Σ**

**σ**

**Σ**

**σ**

S_3_

S_1_

S_2_

**Σ**

**σ**

S_3_

S_1_

S_2_

**Σ**

**σ**

S_3_

S_1_

S_2_

**a**

**b**

**c**

**d**

**Π**

**Π**

**Π**

**Π**

Fig. S2

**S3. Propagation stability of vector fields carrying the arbitrarily tunable OAM**

In Fig. S3, the top row shows the measured intensity pattern of the scalar vortex top-hat field with the helical phases of exp(+*j*20*φ*), which undergoes an evolution from the top-hat profile at *z* = 0 to the multi-ring structure. In the middle row, for the vector field created by a pair of orthogonal polarized bases with the helical phases of exp(±*j*20*φ*) when *T* = 0.32, its propagation behavior has no difference from the scalar vortex field (the top row), implying that the vector field is propagation stable. For the vector field created by a pair of orthogonal polarized bases with the helical phases of exp(+*j*20*φ*) and exp(−*j*5*φ*) when *T* = 0.32, the initial vector field at *z* = 0 will separate into two scalar fields at *z* = 1.2 m, implying that this vector field is unstable during its propagation, as shown in the bottom row. In particular, in the plane *z* = 1.2 m, the measured intensity pattern in the bottom row includes the more rings over the measured patterns in the top and middle rows. This indicates that the input vector field at the plane z = 0 will separate into two scalar vortex fields with the respective helical phases of exp(+*j*20*φ*) and exp(−*j*5*φ*). The ring shown by the red arrow belongs to the scalar vortex field of exp(+*j*20*φ*), while the rings shown by the green arrows belong to the scalar vortex field of exp(−*j*5*φ*). Although the azimuthal intensity still keeps the homogeneity, the local OAM is nonuniform (the OAM is 20*ħ* per photon in the ring shown by the red arrow, while the OAM is −5*ħ* per photon in the rings shown by the green arrows). Therefore, the vector field created by a pair of scalar vortex fields with the helical phases of exp(+*jmφ*) and exp(−*jm*′*φ*) when *m* ≠ *m*′ is unstable during its propagation.

0

1.2 m


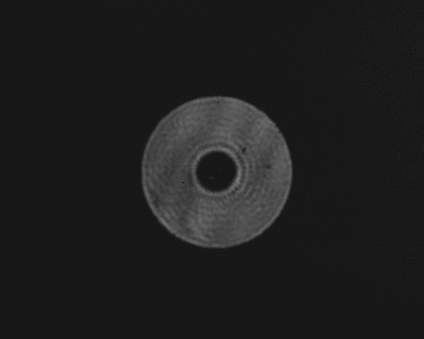

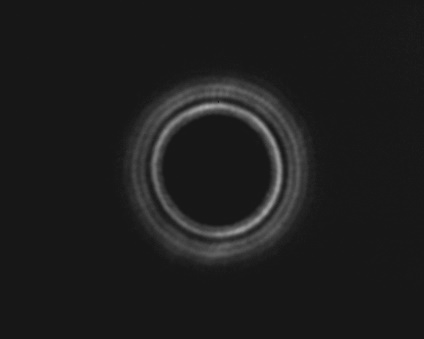

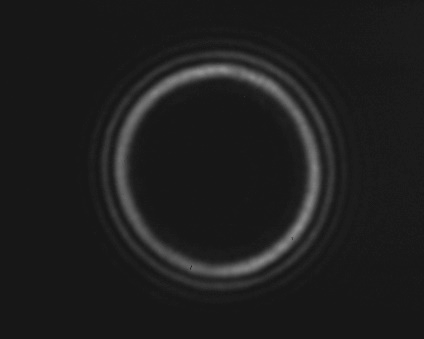

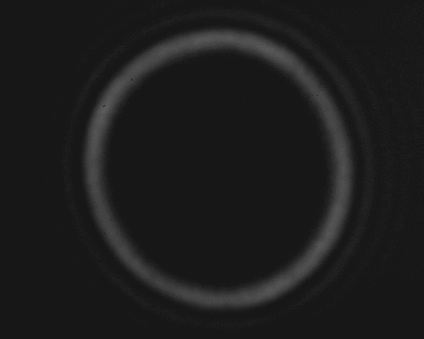

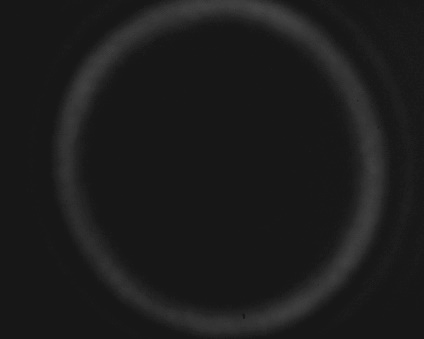

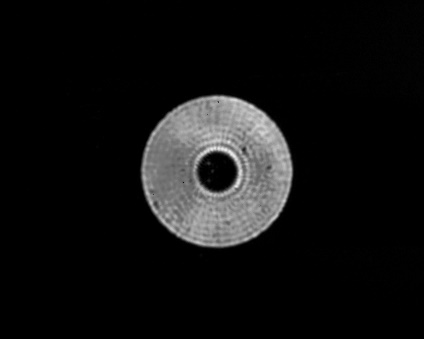

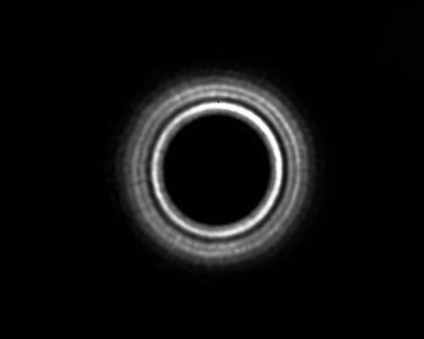

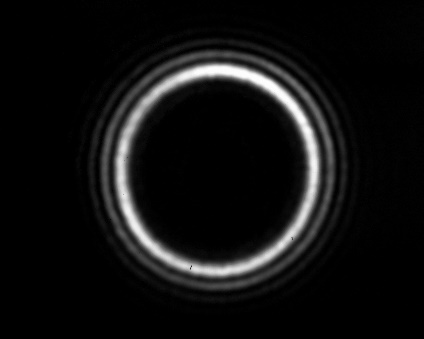

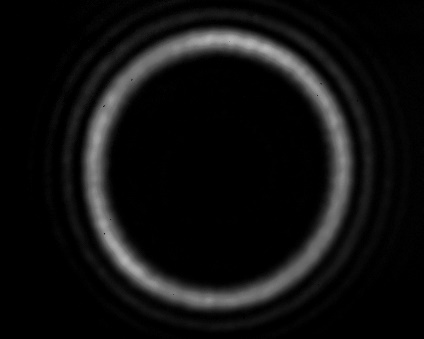

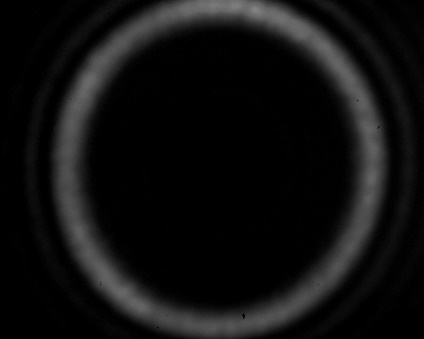

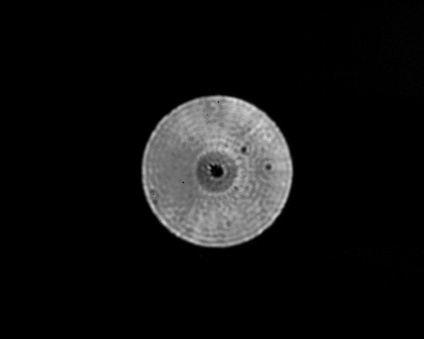

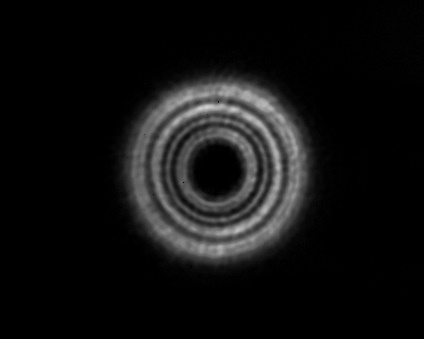

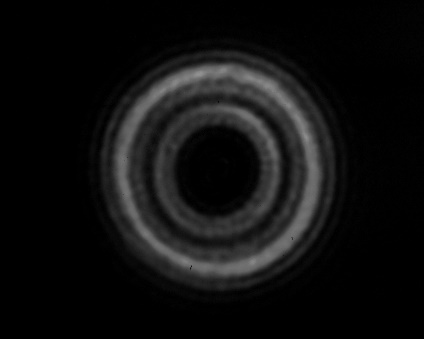

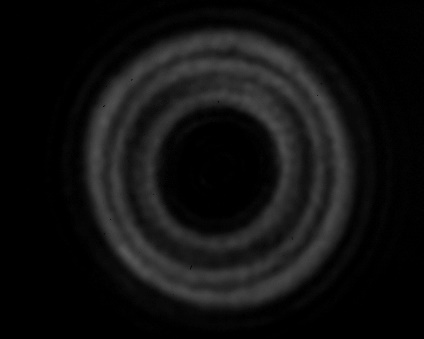

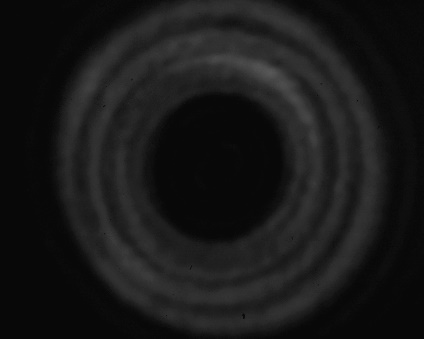


exp(+*j*20*φ*)

exp(−*j*5*φ*)

Fig. S3

**S4. The orbital motion of trapped particles**

For a vector field described by Eq. (6) in the main text, the OAM per photon is . When the focused vector field is used to trap the particles and then drive the orbital motion of the particles, the power density *P* irradiating the particles should be in direct proportion to *P*_0_/*R*, where *P*_0_ and *R* are the total power of the vector field and the radius of the principal ring of the focused top-hat vector field, respectively. The angular momentum flux acting on the particles is in direct proportion to *m_eff_P*_0_/*R* instead of *m_eff_P*_0_/*R*^2^, because the diameter of the particle we used is larger than the width of the focal ring [Phys. Rev. Lett. **90**, 133901 (2003)]. Therefore, the azimuthal momentum applied on the particles should be in direct proportion to *m_eff_P*_0_/*R*^2^. Based on the law of momentum conservation, the peripheral velocity *v* should be $v\propto m_{eff}P_{0}/R^{2}$ and then the angular velocity *ω* should be $\omega\propto m_{eff}P_{0}/R^{3}$. As a result, the period *τ* of the orbital motion of the trapped particles should be $\tau=1/\omega\propto R^{3}/m_{eff}P_{0}$.

For a vector field with a given topological charge *m*, the period of the orbital motion of the trapped particles has the relationship to the relative intensity fraction *T* as $\tau=1/\omega\propto R^{3}/m_{eff}P_{0}=(1+T)/(1-T)(R^{3}/mP_{0})\propto(1+T)/(1-T)$, which corresponds to Fig. 4(b) in the main text.

For a vector field with a given *m_eff_* or a given OAM , which can be achieved by different combinations of *m* with *T*, the period of the orbital motion of the trapped particles has the relationship to the radius *R* of the principle ring of the focused vector field with the topological charge *m* as $\tau=1/\omega\propto R^{3}/m_{eff}P_{0}\propto R^{3}$. In particular, we have confirmed that the radius *R* of the principle ring of the focused vector field is approximately in direct proportion to the topological charge *m*. Therefore, for a vector field with a given *m_eff_* or a given OAM , the period of the orbital motion of the trapped particles has the relationship to the topological charge *m* as $\tau=1/\omega\propto R^{3}/m_{eff}P_{0}\propto m^{3}$, which corresponds to Fig. 4(c) in the main text.

As examples, Fig. 4(a) shows the time-lapse photographs of the orbital motion of the trapped particles, driven by the focused vector fields with a fixed |*m*| = 16 for three different fractions of *T* = 0, 0.1 and 0.3 (see also Video). In the 1st row for the vector field with *m* = 16 and *T* = 0, the trapped particles move clockwise around the ring focus with an orbital period of *τ*~2.47 s. In fact, in this case the vector field degenerates into the scalar vortex field with *m* = 16. When *T* is increased to *T* = 0.1, as shown in the 2nd row, the trapped particles move still clockwise around the ring focus but the orbital period increases to *τ*~2.94 s. When *T* is further increased to *T* = 0.3, as shown in the 3rd row, the orbital period of the trapped particles further increases to *τ*~4.75 s. When *m* is switched from the positive one (*m* = 16) to the negative one (*m* = −16) when keeping *T* = 0.3, the motion direction of the trapped particles is synchronously reversed with an orbital period of *τ*~4.99 s. It should be pointed out that for the vector fields with *T* = 1 (corresponding to the hybridly polarized vector fields reported in Ref. 28), no orbital motion of the trapped particles is observed, implying that such a kind of vector fields carry no OAM. Figures 4(b) and 4(c), plots the dependence of the period *τ* of the orbital motion of the trapped particles, on *T* for different *m* and on *m* for different *m_eff_*, respectively. Clearly, the symbols (experimentally measured orbital periods) are in good agreement with the fitted curves by the formula $\tau\propto R^{3}/m_{eff}$ (where *R* is the radius of the principal ring of the focusing field and $R\propto m$).

**S5. Video of orbital motion of trapped particles**

A video of orbital motion of trapped particles driven by the focused vector fields with a fixed |*m*| = 16 for three different fractions of *T* = 0, 0.1 and 0.3.
